# Research progress on cancer-associated fibroblasts in osteosarcoma

**DOI:** 10.32604/or.2024.054207

**Published:** 2025-04-18

**Authors:** LIWEN FENG, YUTING CHEN, WENYI JIN

**Affiliations:** 1 Department of Oncology, Beijing Chao-Yang Hospital, Capital Medical University, Beijing, 100020, China; 2 Cancer Center, Union Hospital, Tongji Medical College, Huazhong University of Science and Technology, Wuhan, 430022, China; 3 Department of Pathology, Peking Union Medical College Hospital, Chinese Academy of Medical Sciences & Peking Union Medical College, Beijing, 100730, China; 4 Department of Orthopedics, Renmin Hospital of Wuhan University, Wuhan, 430022, China

**Keywords:** Osteosarcoma (OS), Cancer-associated fibroblasts (CAFs), Tumor microenvironment (TME), Bone tumor

## Abstract

Osteosarcoma (OS) is a prevalent primary bone malignancy with limited treatment options. Therefore, it is imperative to investigate and understand the mechanisms underlying OS pathogenesis. Cancer-associated fibroblasts (CAFs) are markedly abundant in tumor stromal cells and are essentially involved in the modulation of tumor occurrence and development. In recent years, CAFs have become a hotspot as researchers aim to elucidate CAF mechanisms that regulate tumor progression. However, most studies on CAFs are limited to a few common cancers, and their association with OS remains elusive. This review describes the role and current knowledge of CAFs in OS, focusing on their potential cellular origin, classification, and diverse functionality. It was found that CAFs influenced OS tumor cell signaling, proliferation, invasion, metastasis, epithelial-mesenchymal transition, stemness maintenance, angiogenesis, and the ability to modify immune system components. Furthermore, findings on other common cancers indicated that effective therapeutic strategies included the manipulation of CAF activation, targeting CAF-derived components, and depletion of CAFs by biomarkers. This review provides new insights and a theoretical basis for OS research.

## Introduction

Osteosarcoma (OS) accounts for 35% of primary bone malignancies. It has been estimated as the 3rd most common cancer in children and adolescents after lymphomas and brain tumors, and can substantially affect the health of patients [1,2]. The prognosis of OS is poor with approximately 3600 new cases and 1720 deaths reported in the USA in 2020 [3]. Furthermore, the 5-year survival rate of metastatic OS patients is only around 30%, and intense chemotherapy and surgery can cause long-term toxicity and reduced quality of life even in localized OS patients [4]. However, the current treatment strategies for OS have been significantly limited and not advanced in the past five decades [5–8]. Although many advanced solid tumor patients benefit from cancer-targeted therapies and immunotherapies, targeted gene mutations in OS remain controversial, and current immunotherapeutic strategies rarely stimulate an immune response against OS and thus have minimal benefit. Moreover, in OS, the significantly rearranged genome lowers the possible use of candidate targets. In addition, the OS tumor microenvironment (TME) has complex and detrimental functions, inducing cytotoxic T-lymphocytes (CTLs) apoptosis and promoting OS cell’s escape from immune surveillance, thus establishing an immunosuppressive TME. Therefore, in this review, an alternative approach was taken by shifting the focus to TME stromal cells rather than OS tumor cells.

Recent studies have indicated that tumor occurrence and progression are not only modulated by tumor cells but also by their interaction with TME [9,10]. TME primarily comprises the extracellular matrix (ECM) and surrounding heterogeneous cells that may include vascular endothelial cells, immune cells, macrophages, and cancer-associated fibroblasts (CAFs). CAFs are a predominant component of TME (>50% of stromal cells) and are essential for ECM formation. The sustained activation of CAFs is associated with the abnormal fibrosis of tumor tissues [11,12]. In recent years, CAF’s malignant phenotypic switch has been linked with the development, invasion, metastasis, and drug resistance of tumors [13,14]. Furthermore, the association of CAFs with OS occurrence and development has attracted much attention. Several single-cell sequencing studies have confirmed the presence of CAFs in OS [15,16]. In 2022, Huang et al. performed a single-cell transcriptome sequencing [15] to examine 3 paired primary, recurrent, and lung metastatic lesions in OS patients, and carried out a bioinformatic analysis of the GSE152048 and GSE162454 datasets of the Gene Expression Omnibus (GEO) database. The results indicated that in OS, CAFs had a relatively high infiltration level and markedly enriched the epithelial-mesenchymal transition (EMT) pathway [15].

This review describes the role and current knowledge of CAFs in OS, primarily focusing on the origin and heterogeneity of CAFs in OS, their association with tumors and other stromal cells, and their potential use as therapeutic targets. Furthermore, this review also discusses the functions of CAFs in OS progression, such as proliferation, invasion, metastasis, EMT, stemness maintenance, angiogenesis, and the ability to modify immune system components, as well as their underlying mechanisms. Moreover, CAF’s association with other cancers is also summarized to comprehensively understand CAF’s biology.

## Origins and Phenotypes of CAFs

### CAF’s origins and activation pathways

The CAFs can be simply defined as fibroblasts located within or adjacent to tumors. Furthermore, they are identified by their spindle-shaped morphology, lack of lineage markers in epithelial, endothelial, or immune cells, and the absence of common mutations in cancer cells [17]. CAFs mainly originate from normal or resting fibroblasts, which are located in normal tissues or have not been activated by tumors, etc., as well as mesenchymal stem cells (MSCs), pericytes, adipocytes, and endothelial cells [11,18]. Studies on CAF origin in OS are primarily focused on bone marrow MSCs (BM-MSCs) and normal fibroblasts [19–23].

In TME, developed BM-MSCs can be activated to form α-smooth muscle actin (α-SMA) expressing myofibroblasts, which are then considered CAFs [12]. Pietrovito et al. indicated that OS cells induce growth-regulated oncogene-α (GRO-α), monocyte chemoattractant protein-1 (MCP-1), and transforming growth factor-β1 (TGF-β1), which can recruit BM-MSCs, *in vitro* [24]. During recruitment, the BM-MSCs indicated increased α-SMA and collagen I-α1 expression and improved contractility, suggesting that they transformed into CAFs in an OS cell culture environment. In another *in vitro* study, Lin et al. demonstrated that BM-MSCs could be stimulated to transform into CAFs using the conditioned medium of a U2OS cell line derived from OS [25]. Furthermore, they performed transcriptome sequencing and molecular analysis, which revealed that U2OS markedly induced interleukin 6 (IL-6) overexpression and STAT3 phosphorylation in BM-MSCs, whereas inhibited the IL-6/STAT3 signaling pathways, thereby inducing the U2OS-mediated phenotypic switch to CAFs. Moreover, the Notch and Akt signaling pathways have also been linked with the phenotypic switch of BM-MSCs to CAFs [20]. In this study, the OS-derived cell lines, MG-63 and U2OS, can significantly increase expression of the Notch pathway downstream effectors Hes1 and Hey1, as well as Notch-mediated Akt phosphorylation, in BM-MSCs, which promoted their transformation to CAFs [20]. In addition, the whole-genome sequencing of MSCs co-cultured *in vitro* with cell lines of breast cancer, glioma, and pancreatic cancer has indicated similar gene expression profiles between the MSCs induced *in vitro* and tumor CAFs *in vivo*, with both differing significantly from normal MSCs [26]. This study provided evidence that CAFs originate from MSCs.

The CAFs also originate from the normal fibroblasts in the OS microenvironment. An *in vitro* study revealed that the extracellular vesicles (EVs) secreted by OS cells promoted the differentiation of normal lung fibroblasts to CAFs [21]. Mazumdar et al. [21] indicated that human lung fibroblasts treated with EVs from OS cells had increased invasion and expression of α-SMA and fibronectin-1, suggesting that in the OS microenvironment, normal fibroblasts can be activated and transformed into CAFs. This might depend on TGF-β as a marked increase is observed in the phosphorylation of SMAD2, a downstream effector of TGF-β. Moreover, administration of the TGF-β1 receptor inhibitor SB-431542, or CRISPR-Cas9 promoted TGFB1 gene deletion, which significantly interfered with the differentiation of normal fibroblasts into CAFs. Lin et al. employed the saponin-mediated cargo-elimination strategy and effectively eliminated OS-derived EVs to disrupt the communication between OS-EVs and normal lung fibroblasts, thereby reducing the production of CAFs [27]. Another study revealed that OS cell’s exosomal collagen type VI alpha 1 (COL6A1) could transform normal fibroblasts into CAFs with increased migration, contractility, secretion of pro-inflammatory cytokines such as IL-6/8, and activation of the NF-kB signaling pathway targeting IL-6/8 [28]. Salvatore et al. established co-cultured normal fibroblasts with an OS cell line, MG-63, and revealed that the co-culture enhanced MG-63 migration *in vitro* [29]. In this co-culture system, fibroblasts had altered morphology and growth patterns, as well as high expression levels of some matrix remodeling proteins such as human cartilage glycoprotein 39 (also known as YKL-40), vascular endothelial growth factor (VEGF), and matrix metalloprotease 1 (MMP1) [29]. However, the authors did not evaluate CAF’s molecular markers in the co-cultured fibroblasts. The literature has indicated that in breast cancer, c-Myc-activated tumor cells stimulate the transformation of normal fibroblasts to CAFs via the insulin-like growth factor (IGF)/IGF-1 receptor axis, thereby providing novel insights into OS research [30].

**Figure 1:**
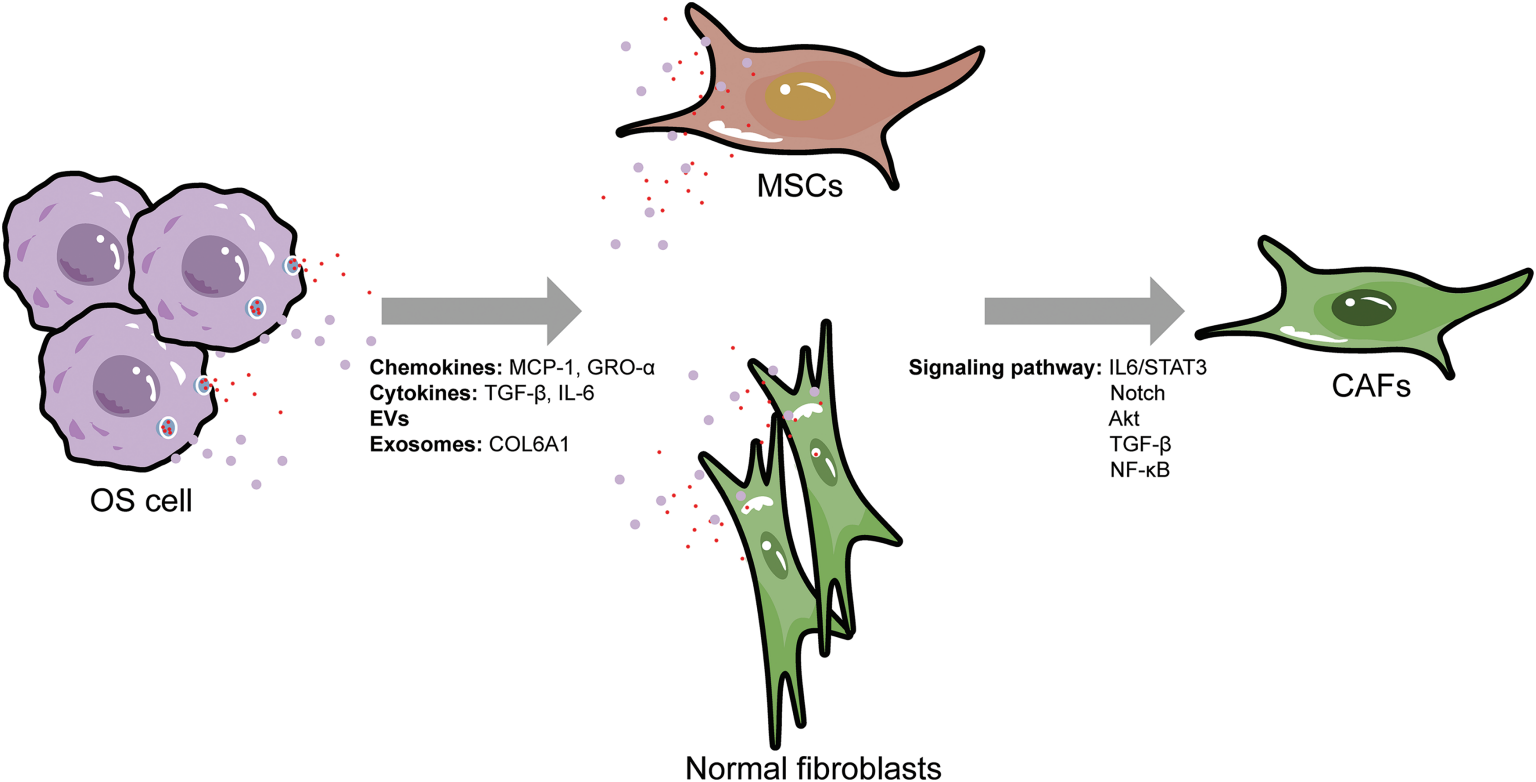
Origins and activation pathways of cancer-associated fibroblasts in osteosarcoma. OS, osteosarcoma; MSCs, mesenchymal stem cells; CAFs, cancer-associated fibroblasts; MCP-1, monocyte chemoattractant protein-1; GRO-α, growth-regulated oncogene-α; TGF-β, transforming growth factor-β; IL-6, interleukin 6; EVs, extracellular vesicles; COL6A1, collagen type VI alpha 1.

The differentiation of BM-MSCs and normal fibroblasts into CAFs are complex multiple-step biological process with intricate cell communication networks and various cytokines, which can serve as targets for OS therapy and warrant further research (Fig. 1).

### Phenotypes and markers of CAFs

CAFs are constantly activated in TMEs, and some classical markers of CAFs are associated with the proliferation, contractility, and activation of fibroblasts, such as platelet-derived growth factor receptor-α/β (PDGFRα/β), α-SMA, vimentin, caveolin-1 (Cav-1), fibroblast activation protein (FAP), and desmin [31]. Table 1 summarizes well-recognized CAF markers. However, these classical markers are not CAFs-specific and are also expressed by other cell types. In addition, some CAFs may not express all of these markers simultaneously and indicate heterogeneity based on their specific TMEs or unique subpopulation functions. Moreover, several studies have suggested that CAFs have heterogeneous functions, transcriptional profiles, TME subtypes, and spatial relationships with tumor cells [32–34]. Therefore, the identification and detection of multiple cell surface markers can be useful for the classification of subpopulations and the investigation of CAFs.

**Table 1:** Biomarkers of cancer-associated fibroblasts

Biomarker	Abbreviation	Function
Alpha smooth muscle actin	α-SMA	Cell contraction, cell structural integrity, connective tissue growth
Fibroblast specific protein-1	FSP-1/S100A4	Cell motility and tissue fibrosis
Fibroblast activation protein	FAP	Extracellular matrix remodeling, fibrosis, and serine protease activity
Vimentin	Vimentin	Cell motility and cell structural integrity
Platelet-derived growth factor receptor α/β	PDGFRα/β	Receptor tyrosine kinase activity
Caveolin-1	Cav-1	Cell structure, cell signal, and transport
Desmin	Desmin	Intermediate filament subunit and devices that maintain cell motility
Tenascin C	TNC	Extracellular matrix protein, strengthening connective tissue, and cell morphology
Periostin	Periostin	Deposition of extracellular matrix proteins
	CD90/Thy-1	Cell matrix communication and cell signal
Podoplanin	PDPN	Cell motility and adhesion
Integrin β1	Integrinβ1	Cell motility and adhesion
Human C5a receptor	GPR77	Complement activation and pro-inflammatory signal
	CD70	T cell function regulation
	CD10	Metalloendoprotease
	CD74	MHCII chaperone and protein transport
	CD146	Cell adhesion

CAF heterogeneity and subpopulations have been investigated in pancreatic, breast, lung, and colorectal cancers. For instance, in pancreatic ductal adenocarcinoma (PDAC), Öhlund et al. identified two spatially separated CAF subtypes, inflammatory CAFs (iCAFs) and myofibroblastic CAFs (myCAFs), via a genetically engineered mouse model and organoid culture technique [34]. The myCAFs were present near the tumor cells and characterized by α-SMA+, FAP+, and expression of TGF-β response genes. Whereas, iCAFs were located distantly from the tumor cells and characterized by low α-SMA expression and secretion of inflammatory mediators including IL6, C-X-C motif chemokine ligand 1 (CXCL1), LIF, IL11, and CXCL2. Furthermore, the transcriptome analysis of CAF primary cultures from PDAC patients identified four subtypes of CAFs (subtype A–D) [35]. It was observed that subtypes B and D had myCAFs phenotype and expressed α-SMA and ECM, subtype C indicated some features like iCAFs phenotype and expressed inflammatory mediators and complement components, while subtype A exhibited both myCAFs and iCAFs phenotypes. In addition, antigen-presenting [36], metabolic [37], and complement-secreting [38] CAFs have also been defined.

However, the classification of CAF subpopulations is controversial and varies across different types of cancers. The advancement of single-cell RNA-seq (scRNA-seq) has allowed for the further analysis of cell subpopulations. Bartoschek et al. performed a single-cell transcriptome analysis of tumor stromal cells acquired from the genetically modified breast cancer mouse model to identify functionally and spatially distinct subpopulations of breast cancer CAFs, including vascular, matrix, cycling, and developmental CAFs [39]. They indicated that CAF’s heterogeneity is partially attributed to different precursor cells. Vascular CAFs indicated significantly increased expression of angiogenesis-associated genes, matrix CAFs had increased transcription of ECM-associated proteins, and cycling CAF cells were observed in different cell cycle phases such as M, G2, or S, whereas the developmental CAFs were distinguished by the expression of stem cells related genes (Sox9, Scrg1, and Sox10). Another study divided the CAFs of human breast cancer into four subpopulations (S1–S4) using flow cytometry and six common CAFs markers (FAP, integrin-β1, α-SMA, fibroblast-specific protein-1, PDGFR-β, and Cav-1) [40]. These subtypes indicated variable expression in triple-negative, luminal A, and human epidermal growth factor receptor 2 (HER2) positive breast cancer. Furthermore, pan-cancer CAF subtypes have also been reported. Galbo et al. utilized scRNA-seq data from head and neck squamous carcinoma, melanoma, and lung cancer to establish gene signatures for the identification of five pan-cancer CAFs: myCAFs, desmoplastic CAFs, iCAFs, iCAFs-2, and proliferating CAFs, representing five distinct biological functions [41].

The CAF’s subpopulation studies related to OS are rarely reported. CAF subpopulations in OS have been indicated by two scRNA-seq studies and the expression patterns they revealed offered valuable information [15,16]. Huang et al. used 37 signature genes to cluster cells, and the CAFs cluster indicated substantially increased expression of ACTA2 (α-SMA), COL1A1 (collagen Iα1), and CDH11 (cadherin 11) [15]. Moreover, according to the expression of MKI67 (Ki-67), the Div-cell clusters were isolated from CAFs, OS cells, macrophages, and tumor-infiltrating lymphocytes. The researchers also identified increased proliferation of Div-CAFs, Div-OS, Div-macrophages, and Div-tumor infiltrating lymphocytes. Further analysis revealed a markedly enhanced Div-CAFs infiltrating in recurrent lesions than in the primary lesions (96.42% *vs*. 3.58%), which might be related to the activation of the EMT pathway in recurrent tumors. Zhou et al. [16] performed scRNA-seq on 100,987 cells and identified 11 cell clusters based on typical gene markers and t-distributed stochastic neighbor embedding (t-SNE). The fibroblast cluster was identified based on increased COL1A1 (collagen Iα1), LUM (lumican), and DCN (decorin) expressions [16]. Furthermore, t-SNE was used to identify three CAF subpopulations: fibroblast_1, fibroblast_2, and fibroblast_3. These three subpopulations indicated elevated expression of COL1A1 and LUM; however, their expression profiles differed. The fibroblast_1 subpopulation expressed COL14A1 (collagen XIVα1), suggesting that it was derived from stromal fibroblasts. Fibroblast_2 was positive for DES (desmin) and negative for ACTA2 and COL14A1, suggesting it originated from smooth-muscle-like cells. Whereas, fibroblast_3 had high MYL9 (myosin light chain 9) and LUM levels, was positive for ACTA2, and negative for COL14A1 and DES, indicating a similarity to myofibroblasts. In addition, fibroblast_3 also indicated increased levels of the osteoblast markers IBSP (integrin-binding sialoprotein) and SPP1 (secreted phosphoprotein 1), suggesting that this subgroup may have osteoblast-like function. Zhou et al. [16] further analyzed the composition of the three subpopulations in primary OS, lung metastatic, and recurrent lesions. The results revealed that fibroblast_1 and fibroblast_3 were the main CAFs in primary and recurrent lesions, whereas fibroblast_2 cells were the primary CAFs in lung metastatic lesions (up to 75%). However, both the above studies lack spatial information and comprehensive functional annotation on CAFs. Therefore, the CAF subpopulations need further investigation for a better understanding of OS CAFs.

Liu et al. performed an integrated transcriptomic analysis focused on classifying subtypes of CAFs in OS [42] using the GEO database to construct a single-cell atlas of human OS tumor lesions, and evaluated essential marker genes and potential biological activities of each CAF subpopulation. They identified 5 CAF subtypes (CAFs_0 to CAFs_4) in OS using cluster analysis. The CAFs_0 significantly expressed ECM-associated markers SPP1 and MMP13 and had ECM remodeling function, defined as ECM-related CAFs (eCAFs). CAFs_1 expressed the ribosome-encoding gene RPL7, which is closely related to ribosomal biosynthesis, termed proliferation-related CAFs (pCAFs). The CAFs_2 termed iCAFs subtype overexpressed inflammatory genes CXCL14 and C3. The CAFs_3 specifically expressed the ACAT2 gene, defined as contraction-related CAFs (myCAFs), whereas multiple antigen-presentation-associated genes are markedly expressed in the CAFs_4 or antigen-presenting CAFs (apCAFs) subtype.

## CAFs in the Occurrence and Development of Osteosarcoma

It has been indicated that the key role of CAFs in cancer is the deposition and modification of filamentous ECM that promotes tumor cell invasion [13,43]. With ECM modification, CAFs can increase tumor tissue’s contractility, tension, and hardness. CAFs also release various regulatory molecules to cross-talk with tumor cells and participate in tumor proliferation, invasion, metastasis, drug resistance, metabolic reprogramming, angiogenesis, and immune regulation [14]. The literature has indicated that in OS, CAFs promote tumor progression. However, “tumor-suppressing fibroblasts” have also been identified [44].

The following sections briefly describe the functions and mechanisms of CAFs in OS.

### CAFs regulate osteosarcoma proliferation

It has been proposed that in OS, CAFs promote tumor proliferation via angiogenesis. Yu et al.’s *in vitro* and *in vivo* analyses indicated that hypoxia-inducible factor-1α (HIF-1α) positively regulated OS cell proliferation and angiogenesis, whereas CAFs competitively protected the HIF-1α mRNA 3′-untranslated region (3′UTR) from miR-143-5p by up-regulating the long non-coding RNA (lncRNA) targeting taurine up-regulated gene 1 (TUG1) [45].

Zhao et al. [46] indicated that CAFs release exosomal lncRNA SNHG17, which participates in the molecular cross-talk of OS cells. SNHG17 inhibits miR-2861 by acting as a competitive endogenous RNA, thereby controlling MMP2 expression. This regulatory mechanism contributes to colony formation, increased cell activity, and reduced apoptosis in OS cells both *in vitro* and *in vivo*.

Furthermore, Mahadevan et al. have proposed a mechanism, cell-projection pumping, for the uptake and transfer of cytoplasm between cells [47]. They utilized fluorescence-activated cell sorting and single-cell tracking to elucidate the alterations in fibroblasts and the SAOS-2 OS cell line in a co-culture system. The SAOS-2 cells that acquired fibroblast cytoplasm showed altered morphologies, enhanced migration, and increased proliferation. However, how CAFs affect OS proliferation has not been comprehensively investigated. Whether CAF’s effect on *in vivo* tumor growth is caused by increased cell proliferation or tumor angiogenesis remains unknown, despite *in vivo* and *in vitro* investigations. Therefore, the regulation of OS proliferation by CAFs warrants further research.

### CAFs promote invasion, metastasis, and EMT in osteosarcoma

The most crucial biological features of malignant tumor progression are metastasis and invasion [48]. To acquire invasion and motility properties, epithelial cancer cells undergo EMT, a process that involves the loss of their epithelial phenotype and detachment from the epithelial layer. In the cellular gene expression profile, EMT is characterized by the inhibition of the epithelial markers cytokeratin and E-cadherin, as well as the increase of the interstitial indices N-cadherin and vimentin [48].

In recent years, the effects of CAFs on tumor invasion and metastasis have been widely investigated in various cancers [49,50]. CAFs produce and modify ECM, which then serve as a molecular pathway to facilitate tumor cell migration. Furthermore, CAFs also crucially mediate cell attachment and detachment in a well-organized manner, by modulating the interaction between scaffolding proteins and cell adhesion receptors [51,52]. Huang et al. analyzed the scRNA-seq data to compare the differentially expressed genes in primary and recurrent OS lesions, as well as lung metastatic lesions [15]. The results indicated a markedly activated EMT pathway in lung metastatic and recurrent lesions than in the primary lesions, especially in the CAF cell cluster. This suggests a correlation between CAFs and OS recurrence, metastasis, and EMT activation. Further experiments confirmed elevated expression of CAFs activation markers (α-SMA and FAP) in recurrent OS patients, which were positively and negatively associated with N-cadherin (an interstitial marker) and E-cadherin (an epithelial marker) expressions, respectively. However, they did not investigate the specific molecular mechanisms underlying CAF involvement in EMT activation as well as OS recurrence and metastasis.

The mesenchymal to amoeboid transition (MAT) significantly alters cell morphology and cytoskeleton. Compared to mesenchymal migration, amoeboid migration is much faster and consumes less energy because of the weak dependence on cell adhesion and ECM protein hydrolysis. RhoA-GTP is a key regulator of MAT, while Rac1-GTP uses WAVE2 signaling to decrease actin contractility and, by extension, MAT activity [53,54]. Pietrovito et al. analyzed the BM-MSCs differentiation-induced CAFs *in vitro* and revealed a substantial decrease in Rac1-GTP, an increase in RhoA-GTP bound to OS cells, and a nearly doubled ratio of RhoA/Rac1 [24]. Therefore, CAFs that differentiate from BM-MSCs can promote MAT in OS cells. Furthermore, a cytokine antibody array was also used to detect the soluble molecules secreted by the CAFs-like BM-MSCs [24]. The results indicated increased secretion of IL-6, GRO-α, IL-8, and MCP-1, consistent with the data acquired from ELISA. Moreover, *in vitro* experiments were also carried out using the corresponding antibodies of these cytokines to analyze changes in the invasion and migration patterns of cells with inhibited cytokine activity [24]. It was observed and confirmed that the OS cell’s invasion property was strongly dependent on the aforementioned cytokines, particularly GRO-α and IL-6, while the inhibition of IL-8 and MCP-1 significantly impaired the migration of OS cells.

Zhang et al. analyzed 181 OS patients and found elevated expression of COL6A1 [28]. Furthermore, they also indicated that the correlation between COL6A1 expression and OS pulmonary metastasis was linked with a poor prognosis. COL6A1 normally resides in the ECM and participates in both cell adhesion and collagen remodeling [55]. *In vitro* analyses have confirmed that exosomal COL6A1 stimulates CAFs to secrete increased TGF-β, which further induces COL6A1 expression in OS cells via the SMAD complex (a major regulator in downstream of the TGF-β signaling pathway), thereby promoting EMT and OS cell’s migration [28]. In OS cells, COL6A1 overexpression mediates OS cell-matrix adhesion by increasing the phosphorylation of focal adhesion kinase (FAK) and Src kinase. Moreover, the interaction between COL6A1 and the E3 ligase SOCS5 promoted STAT1 ubiquitination and degradation, thereby mediating OS metastasis [28].

Non-coding RNAs serve as novel and crucial mediators in the exchange of genetic material between cells. Multiple research studies have indicated that OS invasion and metastasis are affected by key non-coding RNAs of CAFs [45,46,56]. Wang et al. used a miRNA microarray to identify 18 miRNAs that were variably expressed between normal fibroblasts and CAFs [56]. Furthermore, they verified that increased miR-1228 expression markedly decreased the suppressor of cancer cell invasion (SCA1), thereby promoting OS cell invasion and migration [56]. Moreover, Zhao et al. demonstrated that CAFs release lncRNA SNHG17, which promotes OS cell migration, possibly by activating MMP2, which may have served as a competitive endogenous RNA sponge for miR-2861 and thus caused the differences observed in the Transwell migration assay [46].

### CAFs help maintain osteosarcoma stemness

In the 1990s, cancer stem cells were extracted from malignant tumors and are a specific cancer cell subset with high self-renewal ability, tumorigenicity, differentiation potential, and drug resistance, which promotes the “stemness” of tumors. Stemness can be regarded as a signature of tumorigenesis, metastasis, drug resistance, and recurrence. CAFs have been observed to promote tumor progression by supporting tumor cell stemness [57]. Moreover, CAFs also secrete elevated levels of chemokine C-C motif ligand 2 (CCL2) to induce the NOTCH1 pathway in breast cancer cells, thereby maintaining their stemness [58]. In addition, in colorectal cancer, they have been reported to activate the Wnt/β-catenin pathway via the exosomal lncRNA H19, thereby maintaining colorectal cancer cells’ stemness [59]. The NOTCH1 and Wnt/β-catenin signaling pathways are essential for stem cell renewal and differentiation, and their aberrant activation is a crucial event in tumor occurrence and tumor stem cell differentiation [60,61]. Zhang et al. studied CAFs and COL6A1 in OS and found that, in addition to the above EMT and metastasis mechanisms, COL6A1 overexpressing CAFs increased the proportion of CD133+ (a stem cell biomarker) cells in OS by secreting TGF-β, suggesting that they promoted the stemness of OS tumor [28].

### CAFs control osteosarcoma angiogenesis

Angiogenesis is critical for tumor occurrence and development because new blood vessel formation provides nutrients and oxygen for cancer cell progression [62]. Recent research has indicated that CAFs essentially promote angiogenesis to meet the requirement of malignant tumor proliferation [63,64]. A previous study revealed that CAF-derived CXCL12 recruits bone marrow-derived endothelial progenitor cells to stimulate neovascularization [65]. Furthermore, CAFs release increased amounts of pro-angiogenic factors including PDGF-C, fibroblast growth factor (FGF), VEGF-A, and MMP9 to stimulate or accelerate angiogenesis in tumor tissues [64,66]. Moreover, CAFs generated and physicochemically modified ECM can indirectly regulate angiogenesis and blood flow in tumors by controlling tumor matrix stiffness, elasticity, and osmotic pressure [63].

Yu et al. revealed that the CAFs-regulated lncRNA TUG1 promotes OS angiogenesis by up-regulating HIF-1α, confirmed *in vitro* in human umbilical vein endothelial cells (HUVECs), as well as *in vivo* in the formation of peritoneal/lung nodules in nude mice [45]. HIF-1α is a hypoxic TMEs-induced transcription factor that is closely linked with tumor metastasis, angiogenesis, and growth [67]. Furthermore, during hypoxia, HIF-1α promotes angiogenic factor VEGF overexpression, along with endothelial cell proliferation, differentiation, and chemotaxis [68]. Pietrovito et al. observed that in OS cells, CAFs-like cells can stimulate the release of VEGF, IL-8, PDGF-BB, and angiopoietin and significantly increase capillary network formation in HUVECs *in vitro* [24]. Zeng et al. found that VEGF-A expression was positively correlated with the CAFs marker FAP, overexpression of which activated the phosphorylation of Akt and ERK in HUVECs, thereby significantly increasing HUVEC’s proliferation rate [69].

Moreover, Salvatore et al. reported increased VEGF expression and angiogenesis stimulation *in vitro* in co-cultured of OS cells and CAFs without endothelial cells [70]. Since anti-angiogenic drugs are crucial for the clinical treatment of OS, antagonizing VEGF secreted by CAFs could inhibit OS angiogenesis.

### CAFs regulate osteosarcoma immunity

CAFs are crucial regulators of tumor immunity [71]. They have various immunomodulatory functions, which fall into three main categories. First, CAFs secrete different cytokines, exosomes, growth factors, and chemokines, which interact with tumor-infiltrating immune cells and other TME immune components and facilitate immunosuppressive TME formation, thus enabling cancer cells’ immune surveillance escape. For example, CAFs secreted IL-6, CXCL12, and MCP-1 promote the polarization of tumor-related macrophages toward the tumor-promoting phenotype M2, thereby impairing effector T cell responses and inducing immunosuppression [72,73]. Second, CAFs and remodeled ECM’s physical properties regulate anti-tumor immunity, because highly cross-linked ECM can act as physical barriers to CD8+ T cell infiltration [74]. Finally, CAFs indirectly affect the recruitment and activity of immune cells by up-regulating the expression of immune checkpoint molecules including factor-associated suicide (FAS), programmed death ligand 2 (PD-L2), and FAS ligand (FASL) [75,76]. These studies suggest that CAFs can process and cross-present tumor antigens through the major histocompatibility complex I (MHC I), to provide redundant connections to the T cell receptors on CD8+ T cells. The abundance of CAFs-expressed FASL induces the apoptosis of FAS-expressing CD8+ T cells, while the PD-L2 expressed by CAFs induces T cell incompetence by interacting with the immune checkpoint molecule programmed cell death protein 1 (PD-1).

Although CAF’s immunomodulatory effects primarily involve immunosuppression and tumor promotion, its regulation of anti-tumor immunity is a double-edged sword that might be linked with the heterogeneity of CAF subtypes or TMEs. Therefore, studies on the CAF-mediated regulation of anti-tumor immunity should consider the influence of CAF subtypes. In melanoma, PDPN+/FAP− CAFs have been shown to stimulate anti-tumor immunity by coordinating the formation of tertiary lymphoid structures of tumors [77]. In this case, PDPN+/FAP− CAFs were positively linked with improved responses to immune checkpoint therapy and survival rates.

Huang et al. analyzed the scRNA-seq data of recurrent OS patients and revealed that lysyl oxidase (LOX) was markedly up-regulated in CAFs, and was positively related to the infiltration level of macrophages (r = 0.209, *p* = 1.24-03) and CD8+ T cells (r = 0.16, *p* = 1.34-02) in the TIMER database [15]. Furthermore, CAFs were treated with LOX inhibitors, and a notable reduction was observed in M2 macrophages (CD163+) levels in an *in vitro* co-culture/*in vivo* subcutaneous xenograft tumor model [15]. This data suggested that the LOX expressed by CAFs could remodel TME and modulate macrophage polarization, highlighting a possible mechanism of OS recurrence and metastasis. Song et al. revealed a positive role of CAFs in anti-tumor immunity [44]. Furthermore, they analyzed data from OS tissue specimens in the TARGET database and identified two TME subtypes using matrix factorization and a genetic classifier: S1 (infiltration type, abundant immune and stromal infiltrates) and S2 (escape type, lack of effective cytotoxic responses and loss of MHC I expression). Moreover, they labeled CAFs with α-SMA and FAP and revealed that the relative CAFs abundance was markedly higher in S1 than in S2. In addition, Masson’s trichrome staining (to assess fibroblasts/fibrosis) and CD8 immunohistochemical staining (to assess CD8+ T cell abundance) were carried out on 47 primary OS patients [44]. The data confirmed that CAFs or fibrosis expression were positively correlated with the abundance of infiltrating CD8+ T cells and that CAFs improved the efficacy of neoadjuvant chemotherapy for OS. However, the aforementioned finding conflicts with other studies suggesting the multifunctionality of CAFs-immune cell interactions in OS TME, and Song et al. [44] have not explained the possible cause for the contradictory results. Therefore, additional molecular studies are needed to confirm the function of FAP+ α-SMA+ CAFs subpopulations in OS.

**Figure 2:**
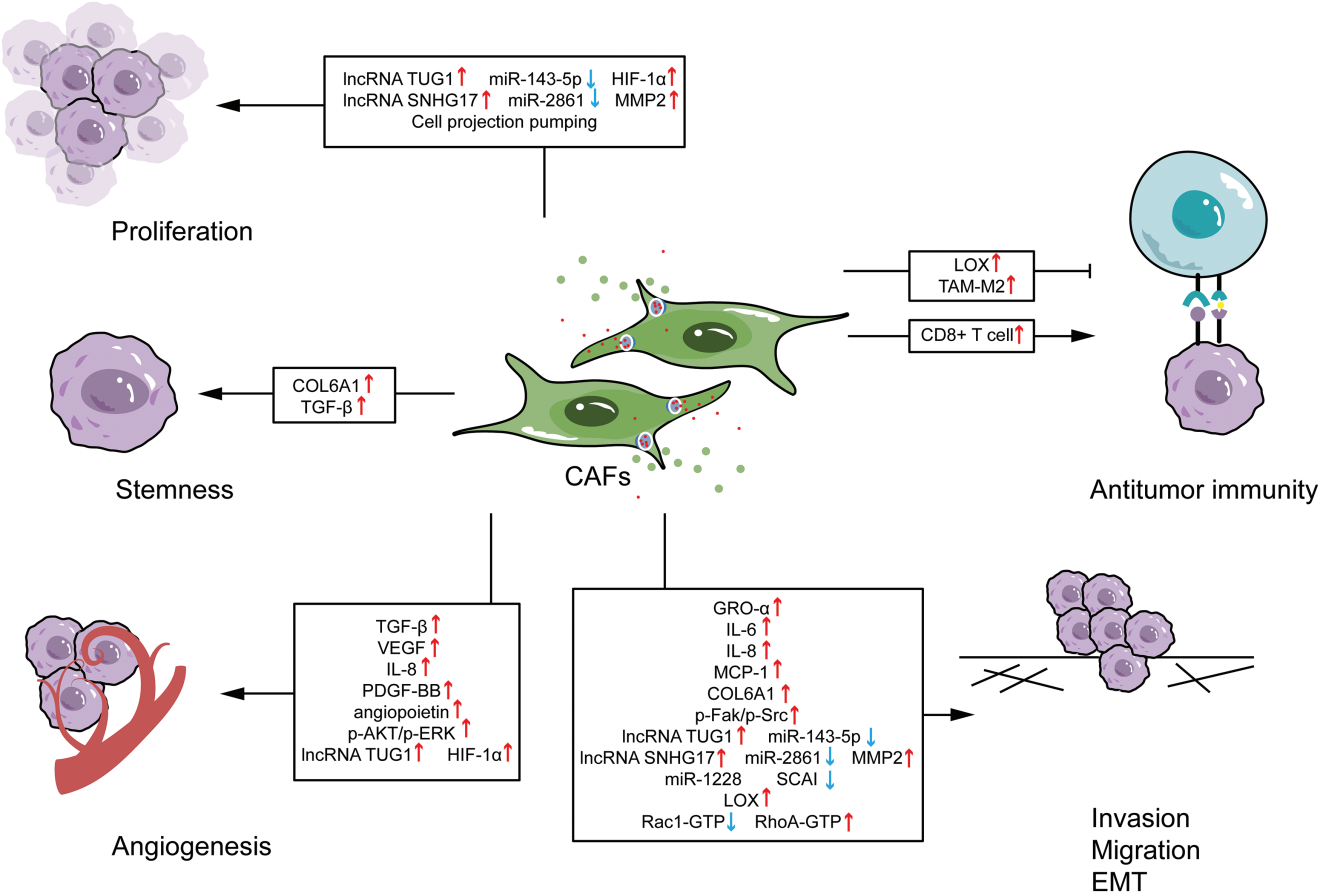
Effects of cancer-associated fibroblasts on osteosarcoma functions. CAFs, cancer-associated fibroblasts; EMT, epithelial-mesenchymal transition; lncRNA, long non-coding RNA; TUG1, targeting taurine up-regulated gene 1; HIF-1α, hypoxia-inducible factor 1 alpha; MMP2, matrix metalloprotease 2; COL6A1, collagen type VI alpha 1; TGF-β, transforming growth factor-β; LOX, lysyl oxidase; TAM-M2, M2-like tumor-associated macrophage; VEGF, vascular endothelial growth factor; PDGF, platelet-derived growth factor; GRO-α, growth-regulated oncogene-α; MCP-1, monocyte chemoattractant protein-1; SCA1, suppressor of cancer cell invasion.

Currently, studies on CAF’s functions in OS are focused on proliferation, invasion, metastasis, EMT, stemness maintenance, angiogenesis, and immunoregulation (summarized in Fig. 2). Although the majority of the aforementioned studies have reported a pro-tumor effect of CAFs in OS, the dual role played by CAFs in OS progression should not be overlooked, especially the positive regulation of anti-tumor immunity. These results highlight that CAFs of different developmental stages and subtypes may have different functions, which require further investigation.

## Pharmacology and Targeting Strategies of CAFs

As outlined in the introduction, the overall treatment response of OS patients has been relatively unsatisfactory for the last five decades. Based on the above-mentioned studies, it can be inferred that CAFs are essentially associated with OS initiation and progression. Compared with OS tumor cells, CAFs feature genetic stability and clear biomarkers, suggesting that targeting CAFs could be a potential therapeutic strategy [7,13]. However, OS-related treatment data or clinical trials directly targeting CAFs are still lacking. Therefore, after the comprehensive review of CAF markers in OS and an in-depth understanding of activation pathways, we propose CAFs as the potential pharmacologic target for future studies and the identification of novel therapies. To comprehensively understand CAFs and their impact on treatments, pan-cancer studies should be reviewed.

### Manipulation of CAF activation

In OS, CAFs are mainly formed by the activation of precursor cells including normal fibroblasts and BM-MSCs [19,21]. Theoretically, therapies should inhibit CAF activation in OS stroma. A characteristic feature of CAF formation is the activation of TGF-β/SMAD2 and IL-6/STAT3 signaling [24,25]. Furthermore, TGF-β has been proven to be a predominant activator of the CAF in OS and multiple tumor species. Schulte et al. reported that compared with other growth factors such as PDGF and FGF, only TGF-β up-regulated CAFs activation-related markers including α-SMA and FAP [78]. Mazumdar et al. indicated that in OS, the conversion of precursors to CAFs is TGF-β-dependent, while TGF-β1 receptor inhibitor SB-431542 or CRISPR-Cas9 mediated TGFB1 deletion can significantly interfere with the CAFs activation [21]. Therefore, the TGF-β/SMAD2 pathway inhibition might be helpful in CAFs-based therapies for OS patients. This strategy is also being evaluated in clinical trials in other cancer types (NCT04524702). However, TGF-β signaling is involved in the complex regulation of many non-stromal cell types and has both the inhibitory or promoting effect on tumors, therefore, it should be carefully targeted and more specific studies are warranted [76].

In addition to targeting growth factor signals such as TGF-β, some intracellular pathway inhibitors have also entered clinical trials for OS treatment [79,80]. It has been observed that Histone deacetylase (HDAC) inhibitors alter epigenetic regulation and intracellular signaling pathways (e.g., JAK1-STAT3) as well as reduce CAF production and activation, thereby inhibiting CAF infiltration into the TME [79]. Mizutani et al. identified that synthetic retinoid AM80 combined with chemotherapy for pancreatic cancer patients could reverse CAF activation in a clinical trial [81]. Moreover, Ferrer-Mayorga et al. found that Vitamin D accumulation prevented normal fibroblasts from converting to CAFs [82]. These studies highlight that CAFs are potential therapeutic targets in OS patients.

### Targeting CAFs derived components

CAFs largely rely on their derived ECM components, cytokines, and exo-substances to promote tumors, therefore targeting these products or altering the CAFs’ secretome is a potential therapeutic strategy. In recent years this strategy has begun a few *in vivo* investigations in OS, in particular the use of nanomaterials to remodel CAFs derived ECM. Wang et al. [83] developed a sequential nanocomposite hydrogel for the controlled release of potent suppressors of CAFs and chemotherapeutic agents, to remodel the CAFs derived ECM thereby overcoming the ECM-induced T-cell exclusion mechanism, which resulted in inducing immunogenic death of OS cells. Hu et al. [84] described a method to disrupt CAFs derived ECM that utilized membrane-anchored and tumor-targeted IL-12-armed (attIL12) T cells bound to other cell surface vimentin, which can interfere with ECM synthesis by CAFs through a complex mechanism such as upregulation of IFN-γ.

However, clinical strategies for targeting CAF-derived components in OS remain largely unexplored, the reports of other cancer types have also provided novel directions. For instance, the primary and most significant chemotactic factor secreted by CAFs is CXCL12 (or stromal cell-derived factor-1, SDF-1), which recruits CXCR4-expressing immunosuppressive Tregs and endothelial progenitor cells, thus participating in tumor angiogenesis, proliferation, and immunosuppression via the CXCL12/CXCR4 axis [65,72,73]. Preclinical data on various malignancies have validated the anti-cancer effects of CXCL12/CXCR4 signaling abrogation [65,85]. Furthermore, it has been observed that smoothened (SMO) hedgehog pathway inhibitors (IPI-926) restrict CAF function by targeting CAF-derived ECM [80,86]. The hedgehog pathway induces fibrogenesis, and SMO on CAF’s surfaces can be activated by sonic hedgehog (SHH, a ligand in the hedgehog pathway) to promote ECM production. SMO inhibitors suppress the ECM-promoting SHH pathway, decrease myofibroblasts levels in the ECM, and increase the tumor’s sensitivity to some chemotherapeutic agents and VEGF inhibitors [80].

### Depletion of CAFs by biomarkers

FAP is an effective marker of CAFs that indicates its active state. ^68^Ga-radiolabeled inhibitor of FAP (FAPI)-PET/CT has been widely used in clinical diagnostic imaging to visualize CAFs in over 20 tumor types [87,88]. Several studies have used FAP to target CAFs and demonstrated the feasibility of targeting CAFs together with early signs of reducing tumor burden [89–92]. Adoptive FAP-specific chimeric antigen receptor (CAR) T-cell therapy is an innovative approach to directly target and deplete most FAP+ CAFs and to restrict tumor stroma production, resulting in antitumor effects [90]. Moreover, therapeutic drugs can also be delivered by certain nanomaterial delivery systems specifically to CAFs via FAP antibody-drug conjugate (e.g., FAP-targeted liposomes, anti-FAP-PE38 immunotoxin), resulting in potent anti-tumor effects [91,92]. Furthermore, despite the success of preclinical strategies, including significant inhibition of xenograft tumor growth in various cancer models, clinical translation is still in the early stages, and some studies have not shown inhibitory activity but rather high toxicity [93,94]. Therefore, the efficacy of CAF-inhibiting strategies by targeting FAP for the treatment of OS is a promising research field.

## Summary and Outlook

This review summarized the origins, activation pathways, phenotypic heterogeneity, and biomarkers of CAFs, and described their effects on tumor progression, as well as their underlying mechanisms, in OS. CAFs are predominant stromal cells in tumor tissues, that modulate various tumorigenic processes, as well as the initiation and progression of malignancy. In addition to OS proliferation, invasion, metastasis, EMT, maintenance of stemness, angiogenesis, and immunoregulation, in other cancers, CAFs have been reported to participate in important biological processes such as metabolic reprogramming, epigenetics, and chemoradiotherapy resistance [13]. However, the involvement of CAFs in OS still requires further investigation. CAFs are heterogeneous cells that show a variety of characteristics and interactions with other cells, which are dynamically altered during tumor progression. This is highlighted by the dual role of CAFs in regulating anti-tumor immunity. Therefore, the identification of CAF subtypes and investigation of their functions are essentially required. To address these issues, simultaneous detection and quantitation of CAF biomarkers can be useful for the identification and repeated evaluation of heterogeneous CAF subpopulations in the future.

Due to their cancer-promoting activities, CAFs have been recognized as a promising therapeutic target for malignancies. To our knowledge, to date, there have been no clinical trials targeting CAFs to treat OS; however, studies on other cancer types can be referred to. In the future, pharmacological research targeting CAFs can be performed based on the following three aspects: inhibition of CAF formation or activation, altering CAFs derived components functionally, and depletion of CAFs by their biomarkers. Furthermore, how to design mediators targeting CAFs is also a promising research area. In recent years, nanoparticles have been widely researched for different treatment applications and have also been employed for OS treatment, however, whether they can be extensively utilized for targeting CAFs in OS deserves further investigation. Overall, this review summarized the current research on CAFs in OS for a comprehensive understanding of the association between CAFs and OS cells, thereby providing a basis for further elucidation of the OS pathogenesis. It was concluded that targeting CAFs represents an effective OS treatment strategy.

## Data Availability

Data sharing not applicable to this article as no datasets were generated or analyzed during the current study.
